# Transabdominal ultrasound sonography findings in peritonitis due to the spread of Crohn's disease to the penetration

**DOI:** 10.1002/ccr3.3215

**Published:** 2020-08-04

**Authors:** Masahiro Takahara, Sakiko Hiraoko, Hiroyuki Okada

**Affiliations:** ^1^ Department of Gastroenterology and Hepatology Okayama University Graduate School of Medicine, Dentistry and Pharmaceutical Sciences Okayama Japan

**Keywords:** computed tomography, penetrating peritonitis, transabdominal ultrasound sonography imaging

## Abstract

In the presence of the clinical signs of peritonitis, transabdominal ultrasound sonography by experienced technicians or physicians could be a fast and reliable imaging tool for confirmation of disease status.

## Graphical abstract

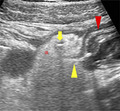

A 31‐year‐old woman came to our hospital because of abdominal pain with peritoneal irritation sign. She was treated for Crohn's disease (CD). Transabdominal ultrasonography (TUS) showed a high peritoneal echo intensity, and a linear hyperechoic image continuous from the thickened small intestine into the peritoneum and a linear hyperechoic image within the peritoneum were also observed (Figure 1). Similar to TUS, computed tomography (CT) showed a small amount of free air and peritonitis adjacent to the thickened small intestine (Figure 2A,B). Based on abdominal and imaging results, we diagnosed the patient with penetrating peritonitis due to CD and after hospitalization, surgery was performed (Figure 3). After surgery, she received a biologic therapy and attained good health without relapse.

**FIGURE 1:**
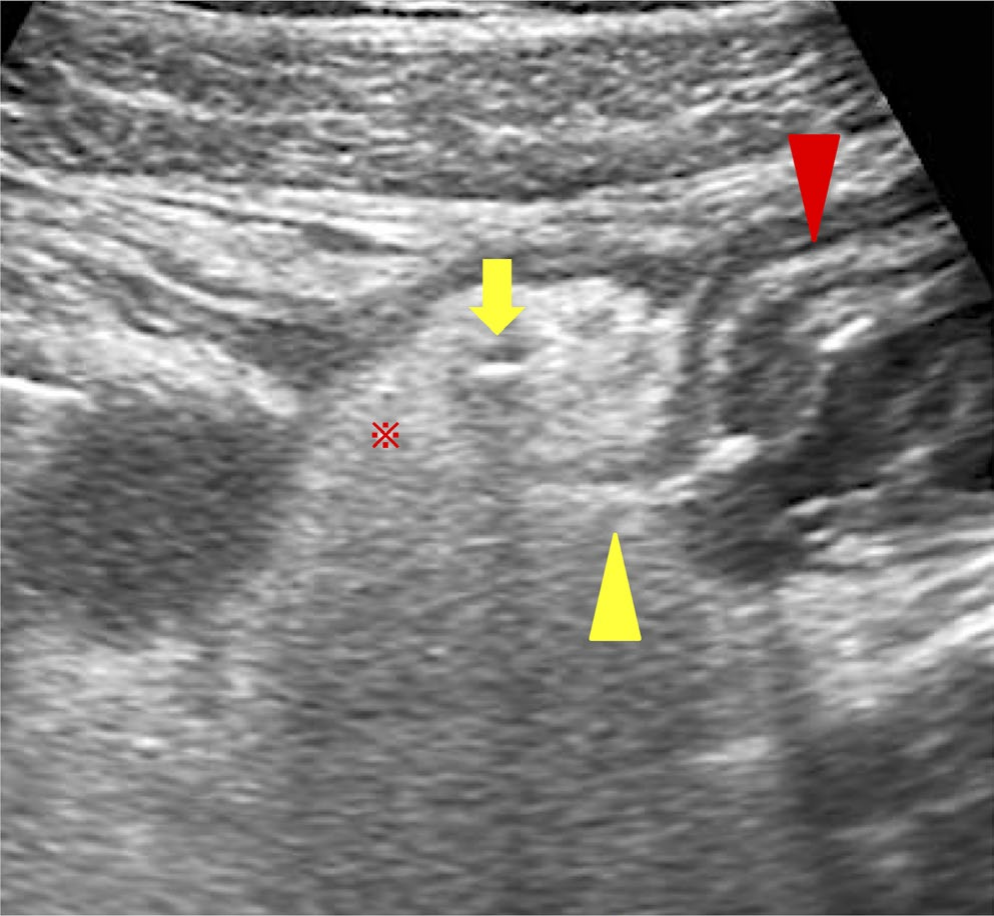
The TUS finding shows a high peritoneal echo intensity (red asterisk), free air (yellow arrow), thickening of the small intestine (red arrowhead), and free air from the penetration site of the thickened small intestine (yellow arrowhead)

**FIGURE 2:**
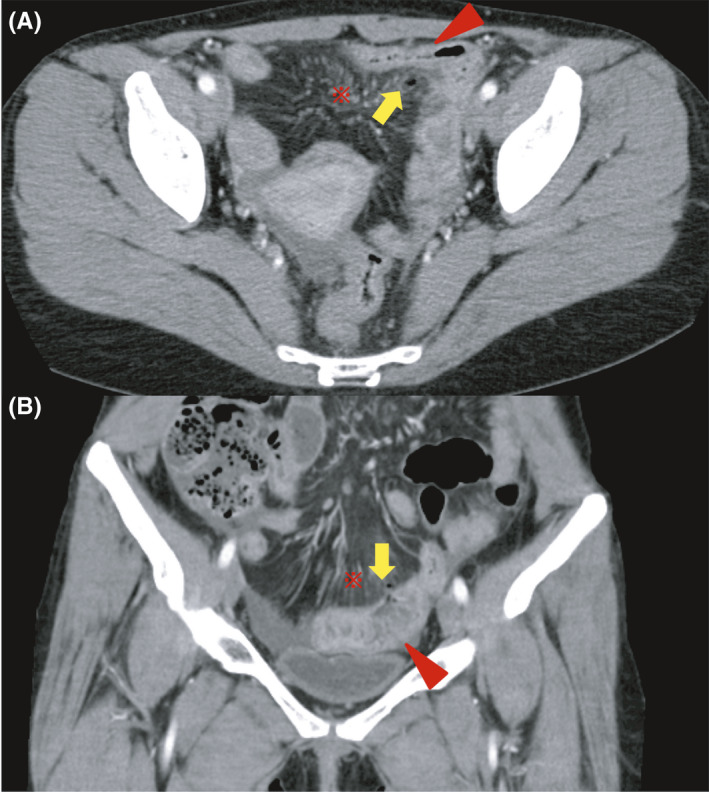
A, B, CT findings showed peritoneal thickening (red asterisk), free air (yellow arrow), and thickening of the small intestine (red arrowhead)

**FIGURE 3:**
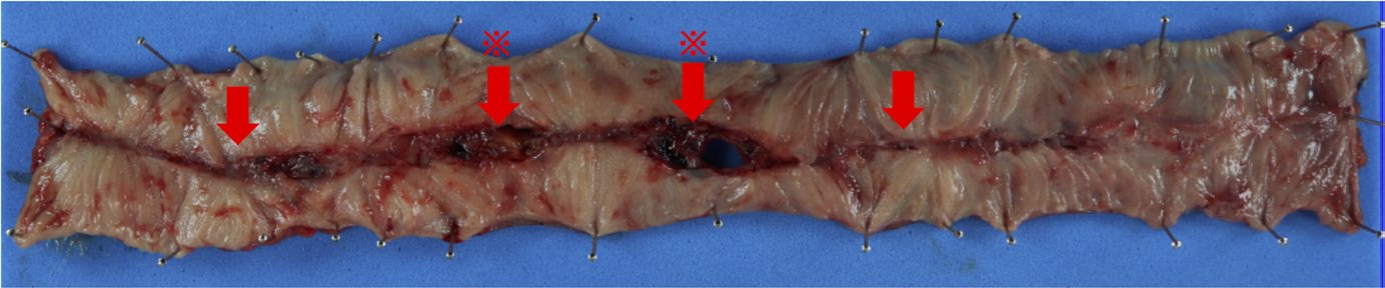
Surgical specimen. The red arrow indicates the longitudinal ulcer and the red asterisk marks the perforation site

The TUS finding of increased peritoneal echo intensity suggests peritonitis. A linear high‐intensity echo suggests free air in the abdominal cavity.^1^, ^2^ A linear hyperechoic image which appeared continuous from the thickened small intestine into the peritoneum suggests the penetration site. This finding was more readily identified than the CT findings. Thus, TUS is a noninvasive, fast, and reliable diagnostic tool that can be evaluated with CT and reveal detailed pathological conditions.

## CONFLICT OF INTEREST

None declared.

## AUTHOR CONTRIBUTIONS

SH and HO: helped and supervised in the writing of the manuscript.
